# Microbial Diversity in Moonmilk of Baeg-nyong Cave, Korean CZO

**DOI:** 10.3389/fmicb.2020.00613

**Published:** 2020-04-23

**Authors:** Shinae Park, Yong-Joon Cho, Da-yea Jung, Kyung-nam Jo, Eun-Jin Lee, Jung-Shin Lee

**Affiliations:** ^1^Department of Molecular Bioscience, College of Biomedical Science, Kangwon National University, Chuncheon, South Korea; ^2^Critical Zone Frontier Research Laboratory, Kangwon National University, Chuncheon, South Korea; ^3^School of Biological Sciences and Research Institute of Basic Sciences, Seoul National University, Seoul, South Korea; ^4^Division of Geology and Geophysics, College of Natural Sciences, Chuncheon, South Korea; ^5^Department of Life Sciences, Korea University, Seoul, South Korea

**Keywords:** limestone cave, speleothem, moonmilk, microbial community, calcite, 16S ribosomal RNA

## Abstract

The Baeg-nyong cave is a limestone cave which has been nominated as the first critical zone observatory (CZO) in South Korea. Moonmilk is a well-known speleothem composed of various carbonate minerals. To characterize moonmilk from the Baeg-nyong cave, we performed mineralogical analyses and applied high-throughput 16S rRNA gene sequencing to analyze the microbial communities, including bacteria and fungi, of dry and wet moonmilk samples. The results showed that the dry and wet moonmilk samples had different and atypical crystal structures, although they were predominantly composed of CaCO_3_. Furthermore, metagenomic data revealed that the dry and wet moonmilk samples collected from an oligotrophic environment had completely different bacterial communities when compared to the outside soil, and there was a difference in bacterial communities even between dry and wet moonmilk specimens. Fungal communities, however, did not differ significantly between dry and wet moonmilk samples. This study is the first metagenomic analysis of two different types of moonmilk with different physical properties and the first report on the microbial diversity of moonmilk from a cave in the first CZO in South Korea.

## Introduction

Many studies have reported on microbial communities and their roles in ecosystems in various environments. A cave is a natural underground opening with no sunlight and a limited supply of nutrients, but that has a stable temperature, high humidity, and high partial pressure of CO_2_ compared to the external environment (Akob and Kusel, 2011). A cave can be divided into four zones according to the amount of light: entrance, twilight, transition, and deep dark zones (Barton, 2006). The entrance zone is the area directly below the entry of the cave. The twilight zone is the area that receives a small amount of sunlight; this zone is occupied by green vegetation to where the sunlight reaches. The transition zone is the area from the entrance to where the dark zone starts. The deep dark zone has very stable physical parameters: no sunlight, relatively low temperature, and relatively high CO_2_ pressure and humidity. The temperature in this zone changes little over the seasons. Therefore, in this oligotrophic environment, microorganisms survive by using alternative pathways, such as ureolysis, ammonification, sulfate reduction, and methane oxidation, rather than photosynthetic activity (Ortiz et al., 2013; Zhu and Dittrich, 2016). Microbial communities in a cave are highly influenced by various factors such as water flow and mineralogical properties (Zhu and Dittrich, 2016). Investigation of cave microorganisms is required to study the cave environment.

A limestone cave contains various types of speleothems, such as soda straw, stalactite, stalagmite, and moonmilk, all of which are mainly composed of calcium carbonate (CaCO_3_). Many studies have demonstrated the role of microbial and physicochemical activities in the formation of CaCO_3_ (Anbu et al., 2016). Moonmilk is a white deposit commonly observed on the walls and ceilings of limestone caves worldwide, that has various textures ranging from soft and powdery to muddy, depending on the water content (Cañaveras et al., 2006; Curry et al., 2009; Cacchio et al., 2012). Additionally, moonmilk is composed of various morphologies with micrometer- or nanometer-length crystals or filaments similar to microbial filaments (Cañaveras et al., 1999, 2006; Hill and Forti, 2007; Curry et al., 2009; Cacchio et al., 2012). Although it remains poorly understood whether moonmilk formation occurs through abiotic or biotic processes, many recent studies have suggested a strong potential for various eukaryotic and prokaryotic activities to influence moonmilk formation (Cañaveras et al., 1999, 2006; Northup and Lavoie, 2001; Barton and Northup, 2007; Portillo and Gonzalez, 2009; Rooney et al., 2010; Portillo and Gonzalez, 2011; Cacchio et al., 2012; Sanchez-Moral et al., 2012). Actively forming moonmilk usually has wet traits, which are known to change to dry traits over time (Borsato et al., 2000). Therefore, studying microbial communities in different states of moonmilk can provide information on the microorganisms involved in moonmilk formation.

The Baeg-nyong cave is a natural limestone cave, which has long horizontal passages from the east to the west, including one main passage and three branches. Some sections of the Baeg-nyong cave, not far from the entrance, are open to visitors for sightseeing, while other sections are strictly restricted. Furthermore, not all, but only certain areas are designated as Korean critical zone observatory (CZO) because of preservation issue. The representative feature of the Baeg-nyong cave is that the path starting from the entrance sharply changes after going straight for about 15 m inward (Woo et al., 2006; Jung et al., 2017). This type of passage can quickly change some of the external factors such as sunlight. In addition, the microclimate in this cave is more stable toward the inside of the cave. As the microbial activity in the Baeg-nyong cave remains unclear, this study aimed to provide the first microbiological information on speleothem in this cave.

Previous studies have evaluated the influence of microorganisms on moonmilk formation using various methods (Cirigliano et al., 2018). However, it remains unclear how the composition of microbial communities differs depending on the type of moonmilk. We performed 16S rRNA gene and ITS next-generation sequencing (NGS) to analyze the microbial community and used scanning electron microscopy (SEM) with energy-dispersive X-ray spectrometry (EDS) and X-ray diffraction (XRD) to examine the crystal morphologies and mineralogical components of moonmilk. We found that the microbial communities differed depending on the type of moonmilk, presenting a variety of bacteria known to deposit CaCO_3_. Furthermore, spheroidal structures were commonly found in dry moonmilk samples, whereas filamentous structures were found in wet moonmilk samples, which may be induced by different microbial communities. This study for the first time investigated the different microbial communities and mineralogical characteristics of moonmilk samples from the Baeg-nyong.

## Materials and Methods

### Study Area

**FIGURE 1:**
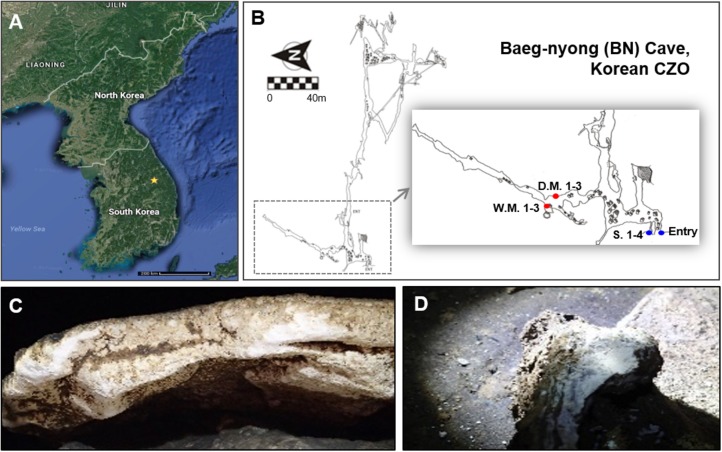
Location of Baeg-nyong cave **(A)** and plain view of this cave **(B)**. Baeg-nyong cave is one of the critical zone observatories (CZO) and located in Pyeongchang-gun, Gangwon-do, South Korea. We collected moonmilk and calcite soil in the first branch and at the entry of the cave, respectively. **(C,D)** Show dry and wet moonmilk colonization on the limestone surface, respectively. The locations where the samples were collected were marked with red (moonmilk) and blue dots (S.: outside soil) on **(B)** (D.M.: dry moonmilk, W.M.: wet moonmilk).

Baeg-nyong cave is located in Pyeongchang-gun, Gangwon-do, South Korea (37° 16′ 19.65′′ N, 128° 34′ 46.03′′ E, 1,875 m) (Figure 1A). Most of the passages, including the entrance zone, have 11.0–13.5°C average temperature and 70–100% of relative humidity. Cave water in the several drip sites near the location of moonmilk formation is alkalescent (pH 8.0).

### Sample Collection and DNA Extraction

Moonmilks on the rock in the middle of the first branch from the entrance were sampled, and this area showed a high pressure of CO_2_ (347–844 ppmv) with clear seasonal changes comparing to the outside (280–533 ppmv). The measurement of CO_2_ pressure was performed between April 2016 and January 2017. Three samples for each moonmilk and four samples for outside soil near the entrance of the cave were collected (Figure 1B). About 1 g of each sample was collected in a 15 ml-tube using a sterile spatula and stored at −20°C until processed. The collection of moonmilk and outside soil samples was performed in October 2016.

DNA extraction from 0.5 g each of moonmilk and soil samples was performed by using FastDNA^®^ SPIN Kit for soil (MP Biomedicals, United States) according to the manufacturer’s instructions. The extracted moonmilk and soil DNA was eluted in 50 μl TE buffer and stored at −20°C.

### NGS Sequencing

To analyze the bacterial community, we amplified the 16S ribosomal RNA gene with universal 341F and 805R primers covering the V3–V4 region (Herlemann et al., 2011). In the case of fungal community analysis, ITS7 and ITS4 primers covering the ITS2 region were used (Ihrmark et al., 2012). PCR reactions were performed as follows for 25 cycles: denaturing at 98°C for 30 s, primer annealing at 55°C for 30 s, and primer extension at 72°C for 30 s. Ramp speed was limited at 1°C/sec during the annealing step to minimize the formation of chimeric sequences. Amplicons were confirmed by electrophoresis and quantified to be normalized to the same amount. Finally, the sequencing library was prepared from 100 ng of the pooled amplicons using NEB Ultra II DNA Library Prep Kit for Illumina (E7645S, NEB, United Kingdom) according to the manufacturer’s recommendation. The sequencing of the amplified product was performed on the Illumina MiSeq (Illumina, United States) machine using the 250 bp paired-end platform.

### Analysis of NGS Data

16S rRNA amplicon analysis was performed by Mothur software v 1.39.5 (Schloss et al., 2009). All reads were trimmed by Trimmomatic 0.36 with default parameters (Bolger et al., 2014). After the removal of adaptors, the forward and reversed reads were merged using PEAR v 0.9.6 and unmerged read, and the read of less than 200 bp were removed (Zhang et al., 2014). The merged reads were aligned by Needleman algorithm, using EzTaxon-e gene database v 2018.05 for bacteria and UNITE database v 7.0 for fungi (Edgar et al., 2011; Kim et al., 2012). Chimeric nucleotides were removed by the uchime program (Edgar et al., 2011). After processed reads were clustered by OptiClust method, operational taxonomic units (OTUs) were extracted according to 97% of nucleotide identity (Westcott and Schloss, 2017). Coverage and diversity index analysis of OTU were performed based on cut off 0.03. Non-metric multidimensional scaling (NMDS) plot analysis and various bar plots were performed by PRIMER-e (New Zealand) and phyloseq package of R and the linear discriminant analysis effect size (LEfSe) tool was used to identify differentially abundant features (Segata et al., 2011; McMurdie and Holmes, 2013).

### X-Ray Diffraction (XRD) and Scanning Electron Microscopic (SEM) Analyses

Every specimen was dried in an oven at 35°C for 5 h before both XRD and SEM analyses. To identify mineral assemblages in moonmilk samples from the Baeg-nyong cave, XRD analysis was carried out through X’pert PRO MPD (PANalytical, Ltd.) housed in the Central Laboratory at Kangwon National University (KNU). Prior to analyzing the samples in the XRD, powder samples were produced using the agate mortar. Generator settings were 30 mA and 40 kV, and anode material was Copper. To perform SEM techniques, all the samples were mounted on the steel stub using the carbon tape. Dehydrated and fixed samples, coated with a thin layer of gold, were examined on CX-200TA (COXEM, Ltd.). The operating condition for SEM image analysis is in the accelerating voltage of 20 kV with a working distance of 8 to 12 mm with an electron generator of the tungsten filament. EDS (EDAX, Inc.) analysis was performed with the SEM examination.

## Results

### Diversity of Bacterial and Fungal Communities in Moonmilk

To investigate the microbial diversity in the Baeg-nyong cave, we collected samples of the various speleothem, including soda straw, stalactite, stalagmite, and moonmilk, as well as cave sediment. However, only moonmilk samples had a sufficient concentration of genomic DNA (10–100 ng/μl per 0.5 g of moonmilk and 50–100 ng/μl per 0.5 g of soil) to perform metagenomic analysis. Moonmilk samples were collected from the first branch of the cave, which is restricted from visitors (Figures 1B–D). Before analyzing the microbial diversity, we categorized moonmilk into two types, dry and wet moonmilk, based on visible traits such as shape and texture. Dry moonmilk was crusty, like flour, and wet moonmilk was soft and sticky, like cream cheese. As wet moonmilk is generally regarded as actively formed, we hypothesized that different microbial communities are dominant in each moonmilk type. In addition, outside soil samples were collected near the entrance of the cave to identify microbial communities specific to the cave environment (Figure 1B).

Next-generation sequencing analysis of the 16S rRNA genes and ITS2 regions was performed to identify the microbial communities of the two types of moonmilk and the outside soil sample. After trimming and chimera filtering, 48,869 reads were used for the analysis of bacterial clusters. The library size of each sample ranged from 1,057 to 10,886, and the total number of OTUs from the whole sample was 1,640 (Supplementary Table S1). Based on estimation of the observed OTU numbers and calculated diversity indices (Shannon index), the bacterial diversity of the outside soil was significantly higher than that of the moonmilk (Table 1). The wet moonmilk samples showed relatively large sample variation in bacterial diversity compared to dry moonmilk samples. Overall, however, these two environments did not differ significantly in bacterial diversity (Table 1). Depending on water availability, different types of bacteria could adapt to the environment and thrive. Additionally, since the elements of cave water are important for the growth of certain microorganisms (Macalady et al., 2008), the presence of moisture in moonmilk may change the microbial composition.

**TABLE 1:** Summary of bacterial community sequencing and diversity indices for each sample.

Type	Sample	Cleaned reads	Observed OTUs	Coverage	Ace	Chao	Shannon
Dry	DM1	6130	270	0.99	302.29	286.38	3.73
	DM2	4999	273	0.99	312.97	292.97	4.13
	DM3	7506	321	1	337.12	328.19	4.12
Wet	WM1	2741	163	0.99	181.15	173.22	3.65
	WM2	10886	331	1	342.64	335.92	4.41
	WM3	4953	191	1	202.97	200.53	3.01
Outside	S1	1057	242	0.92	321	284.13	4.68
soil	S2	1421	271	0.95	320.31	292.03	4.78
	S3	7698	626	0.99	643.73	629.84	4.92
	S4	1478	259	0.96	297.95	275.45	4.62

**FIGURE 2:**
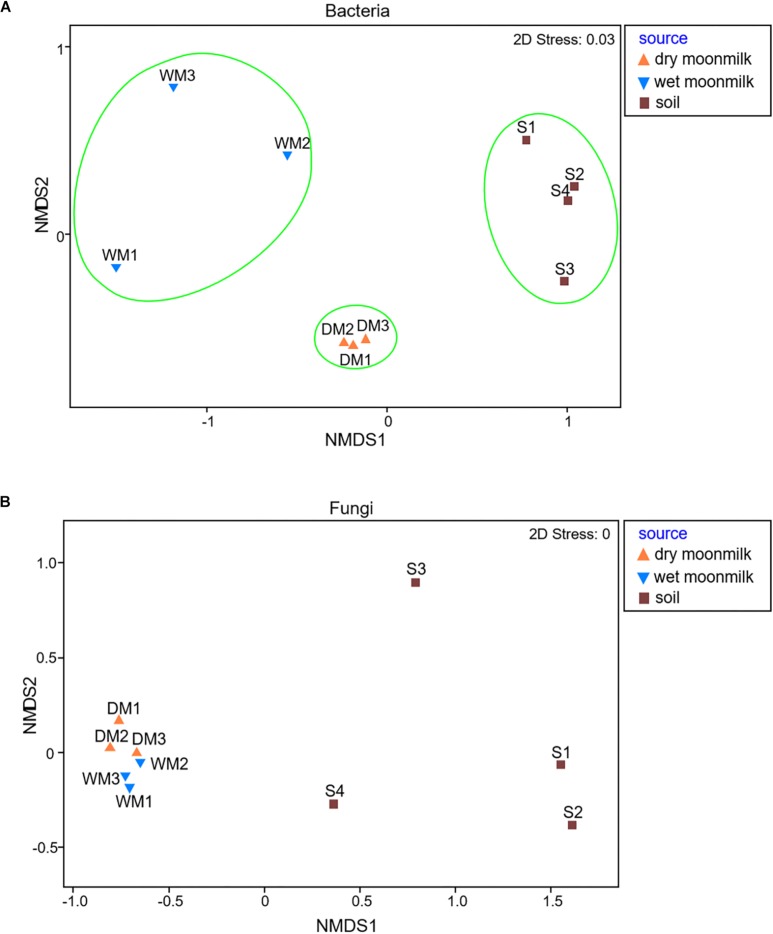
Non-metric multidimensional scaling (NMDS) plots to show microbial communities of moonmilk and outside soil samples for bacteria **(A)** and fungi **(B)**.

Next, microbial communities were compared among samples by non-metric multidimensional scaling (NMDS) to determine their similarity. The results showed that the bacterial communities of the samples were well-grouped depending on their environmental conditions (Figure 2A). The differences in the environmental conditions may have led to differences in bacterial diversity, although the origin of soils and cave speleothem, such as moonmilk, is likely to be a single geologic setting. Even in the same cave environment, specific microbial communities were detected according to the condition of moonmilk.

As for fungi, the library size ranged from 3,980 to 12,985, and 78,103 reads was obtained from the total samples, and 294 OTUs were classified (Supplementary Table S2). The NMDS plot for the fungal communities revealed that they were all grouped together and showed no difference between dry and wet moonmilk samples. This result also confirmed that specific fungal species dominantly occupied the fungal communities of both types of moonmilk (Figure 2B).

### Taxonomic Composition of Microbial Communities in the Two Types of Moonmilk

**FIGURE 3:**
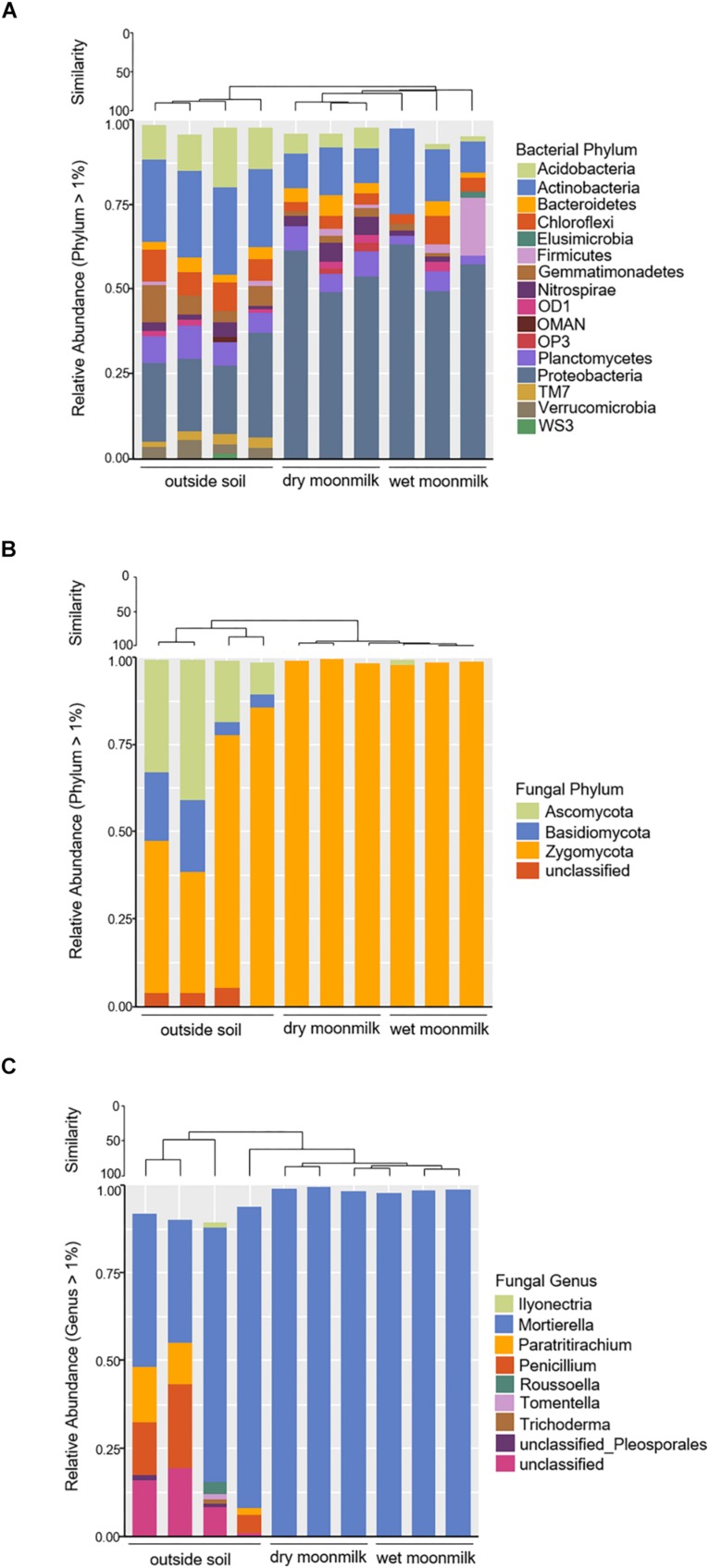
Bar graph representing relative community composition of bacterial phylum **(A)**, fungal phylum **(B)**, and fungal genus **(C)** Which represented ≥ 1% of total sequences by sampling source.

We further investigated the taxonomic composition profile of each sample (Figure 3). Eighteen bacterial phyla showed relative ratios of ≥ 1%. Among them, *Proteobacteria* were the most dominant in dry and wet moonmilks and comprised approximately 54% of the bacterial phyla in the dry moonmilk, followed by *Actinobacteria*, *Acidobacteria*, *Chloroflexi*, and *Bacteroidetes* (Figure 3A). This is similar to the recently reported composition of the microbial community in moonmilk from the Tomba degli Scudi, Tarquinia, Italy (Cirigliano et al., 2018). In contrast, high percentage groups of phyla in the outside soil included *Actinobacteria* and *Acidobacteria*, followed by *Proteobacteria*. The outside soil bacterial composition is similar to that in the temperate rainfall region (Barnard et al., 2013; Kim et al., 2014; Yang et al., 2017).

As for the fungal communities, the diversity indices and fungal genus composition indicated that specific species were present in moonmilk samples (Table 2 and Figures 3B,C). Analysis of the fungal communities demonstrated that *Mortierella* occupied most of the communities. *Motierella* is commonly found in cave soils and has been identified in moonmilk in many caves (Rutherford and Huang, 1994; Garcia-Sanchez et al., 2019).

**TABLE 2:** Summary of fungal community sequencing and diversity indices for each sample.

Type	Sample	Cleaned reads	Observed OTUs	Coverage	Ace	Chao	Shannon	Simpson 0.93	Inverse Simpson
Dry moonmilk	DM1	7525	27	1	33.74	29.63	0.23		1.08
	DM2	6553	18	1	20.2	19.5	0.07	0.98	1.02
	DM3	7340	29	1	31.32	29.6	0.17	0.96	1.05
Wet	WM1	6094	25	1	29.27	29.2	0.19	0.95	1.06
	WM2	7962	41	1	56.21	54.33	0.15	0.96	1.04
	WM3	8414	31	1	78.07	61	0.13	0.97	1.03
Outside	S1	11663	148	1	154.46	151.64	2.43	0.22	4.51
soil	S2	12985	178	1	183.92	179.95	2.58	0.17	5.8
	S3	5587	102	1	104.61	102.71	1.76	0.49	2.05
	S4	3980	53	1	56.46	54.91	0.85	0.73	1.37

**FIGURE 4:**
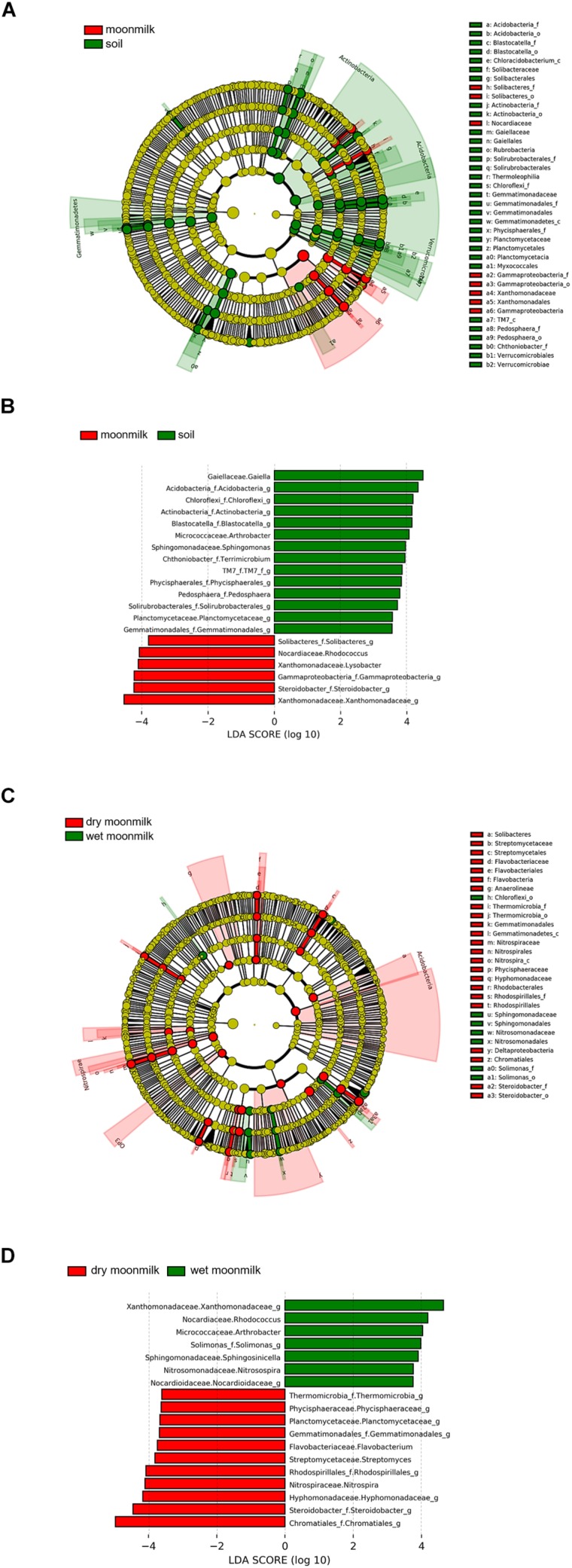
Different abundances of bacterial communities among moonmilk and outside soil. Linear discriminant analysis (LDA) effect size (LEfSe) analysis and visualization were used to select differentially abundant bacterial taxa between moonmilk and soil **(A,B)** and between dry moonmilk and wet moonmilk **(C,D)**. Cladograms show taxonomic levels by rings representing phyla to genera from inside to outside. In bar plots, the LDA score on the x-axis shows log changes in relative bacterial taxa representation.

To identify bacterial species that are specific to moonmilk (Figure 4 and Table 3), we first analyzed the structures of bacterial communities in outside soil and moonmilk. Several genera, such as *Blastocatella*, *Gaiella*, *Arthrobacter*, and *Sphingomonas*, were dominant only in the outside soil samples (Figures 4A,B), whereas some soil-derived bacteria, such as *Rhizobiales* (OTU0003) and *Acidimicrobiales* (OTU0005) were common in all samples (Table 3). Additionally, several genera including *Gammaproteobacteria*, *Steroidobacter*, and *Xanthomonadaceae*, were more abundant in moonmilk than in soil. However, *Xanthomonadaceae* (OTU0016) and *Arthrobacter* (OTU0024) were the dominant genera only in wet moonmilk (Table 3 and Figures 4A,B).

**TABLE 3:** Distribution matrix with the 30 most abundant bacterial taxa among all samples.

OTU number	DM1	DM2	DM3	WM1	WM2	WM3	S1	S2	S3	S4	Order OTU (phylum)
OTU0001	4.14	5.08	2.03	0.18	10.38	40.99	0.76	0.49	0	0.61	Pseudomonadales (G)
OTU0002	24.88	15.16	15.31	0.4	0.22	1.19	0.47	0.14	0.38	0.27	Chromatiales(G)
OTU0003	6.51	4.92	5.61	5.98	2.65	2.48	7.47	5.47	5.74	9.91	Rhizobiales(A)
OTU0004	6.3	4.86	8.66	1.09	1.41	0.46	0.19	0.42	1	0.74	Steroidobacterr-0(G)
OTU0005	2.59	3.7	3.73	3.5	2.03	0.87	2.46	2.24	2.21	2.49	Acidimicrobiales(At)
OTU0006	0.85	1.94	1.21	0.18	0.93	0.67	3.5	4.34	7.98	5.66	EU686603-0(Ac)
OTU0007	4.29	2.72	3.32	1.39	2.02	0.26	0.95	1.4	1.97	1.95	Rhodospirillales(A)
OTU0008	1.09	1.02	2.82	10.98	2.58	1.15	0.19	0.28	0.18	1.13	Gammaproteobacteria-0(G)
OTU0009	0.16	0.02	0.01	7.08	6.39	1.65	0.09	0.07	0	0	Xanthomonadales(G)
OTU0010	0.96	1.3	0.72	14.41	1.24	0.69	1.04	2.52	0.79	1.82	Burkholderiales(B)
OTU0011	0.52	0.24	0.45	0	0.34	0.48	2.65	2.1	7.62	1.21	FJ478799_0(At)
OTU0012	2.3	4.12	0.62	1.28	0.29	0.02	0	0.07	0.05	0.2	Rhodobacterales(A)
OTU0013	0	0	0	0	0.07	14.72	0	0	0	0.34	Lactobacillales(F)
OTU0014	1.99	1.26	1.68	0.47	1.13	0.63	1.7	2.31	1.65	2.02	Planctomycetales(P)
OTU0015	0.07	0.46	0.25	0.55	0.92	0.93	3.31	3.36	3.45	3.64	GQ396871_o(C)
OTU0016	0.05	0	0	1.13	4.67	1.25	0	0	0	0	Xanthomonadales(G)
OTU0017	0.75	1.64	1.07	0.36	2.51	0.04	0.95	0.63	0.06	1.15	Bacillales(F)
OTU0018	1.01	0.6	1.68	0.95	0.38	0.3	2.36	1.47	1.92	1.08	Gemmatimonadales(Ge)
OTU0019	3.43	0.78	2.68	0.11	0.17	0.38	0	0	0.05	0	EU445199_(Ac)
OTU0020	0	0	0.03	0	3.55	1.55	0.38	0	0.25	0.34	GQ396871_o(C)
OTU0021	0.26	1.3	0.87	0.15	0.3	0.44	7.75	3.29	1.14	3.44	Gemmatimonodales(Ge)
OTU0022	1.09	0.76	0.97	0.4	0.25	0.26	1.13	1.33	2.26	0.88	Planctomycetales(P)
OTU0023	0.62	0.8	1.31	0	1.14	1.27	0.95	0.77	0.58	1.08	Cytophagales(Ba)
OTU0024	0.1	0	0	1.61	2.52	2.42	0	0	0	0	Micrococcales(At)
OTU0025	0.39	0.58	0.48	2.92	2.21	0.48	0.09	0	0.03	0	Sphingomonodales(At)
OTU0026	0.51	0.2	0.29	0	0.03	0	3.31	2.8	3.19	2.56	Blastocattella_o(Ac)
OTU0027	0.29	0.52	0.57	0.44	2.23	0.77	0.57	0.7	0.22	0.4	Planctonmycetales(P)
OTU0028	1.04	1.68	1.85	0	0.98	0.1	0.19	0	0.06	0	Streptomycetales(At)
OTU0029	0.47	0.9	1.27	0.04	0.05	0	0.66	0.28	2.22	0.07	GU444092_o(N)
OTU0030	1	1.12	0.57	0.04	0.042	0.16	0.47	1.4	0.97	1.28	Sphingobacterials (Ba)

*A, Alphaproteobacteria; Ac, Acidobacteria; At, Actinobacteria; B, Betaproteobacteria; Ba, Bacteroidetes; C, Chloroflexi; F, Firmicutes; G, Gammaproteobacteria; Ge, Gemmatimonadetes; N, Nitrospirae; P, Planctomycetes.*

When we compared the structures of bacterial communities in dry and wet moonmilks, two taxa of *Gammaproteobacteria* were identified as the two major species in dry moonmilk (OTU0002 and OTU0004) (Table 3). Purple sulfur bacteria (OTU0002), which belong to *Chromatiales*, showed differences in quantity between the two moonmilks; the purple sulfur bacteria accounted for approximately 18.4% of the bacterial community in dry moonmilk, but were rarely detected in wet moonmilk or outside soil (Table 3). *Steroidobacter* (OTU0004) was abundant only in dry moonmilk at an approximate ratio of 4–7% (Figures 4C,D and Table 3). Additionally, genera belonging to *Rhodospirillales* (OTU0007) and *Rhodobacterales* (OTU0012), and the genus *Streptomyces* (OTU0028) were detected specifically in dry moonmilk by LEfSe analysis (Figures 4C,D and Table 3). In wet moonmilk, taxa belonging to the *Xanthomonadales* group (OTU0009) and *Micrococcales* (OTU0024) were mainly detected (Figures 4C,D and Table 3).

### Mineralogical Characteristics of the Moonmilk From Baeg-nyong Cave

**FIGURE 5:**
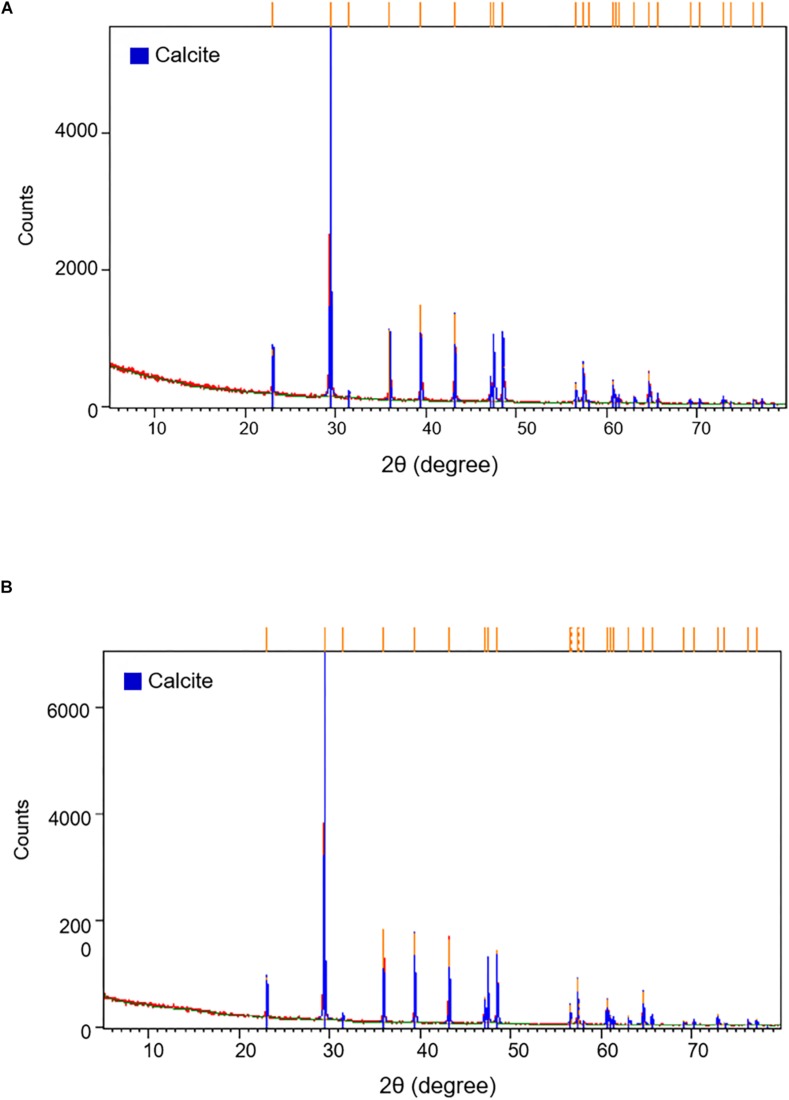
X-ray diffraction pattern of **(A)** dry and **(B)** wet moonmilk samples. XRD patterns demonstrate the intensity (total counts) of a diffracted beam with two times of the incident angle. Orange bars on the top of the graph indicate that the major peaks list of the samples coincided with the calcite crystal (blue bars).

**FIGURE 6:**
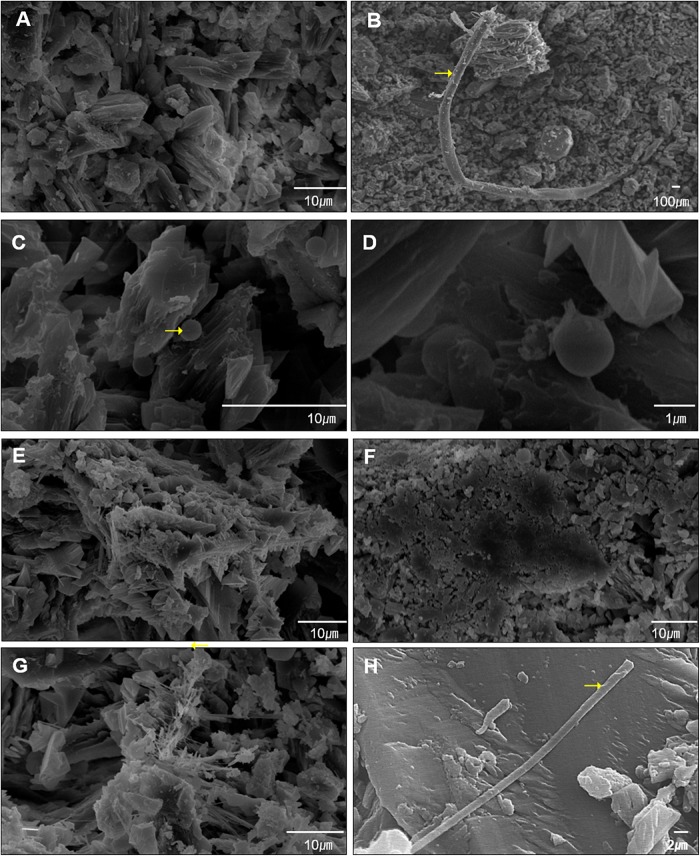
Diverse sizes of filamentous structures were observed by scanning electron microscopy (SEM). **(A–D)** Shows surface of dry moonmilk, **(E–H)** show surface of wet moonmilk. The filamentous structure was quite long in the two moonmilk samples **(B,H)**. Specifically, dry moonmilk contained many cocci structures **(C,D)**. Filamentous structures formed a cluster in moonmilk **(G)**. Yellow arrows indicate the spot for EDS analysis.

Based on the differences in the distinct appearance and microbial community composition, we predicted that different microorganisms within each moonmilk sample interact with each other depending on their environment to affect the various structures of calcite crystal. Information regarding the relationship between calcite morphology and microbial communities may be obtained through mineralogical and crystal morphological analyses. The XRD results showed that both samples of moonmilk were predominantly composed of calcite (Figure 5). SEM images revealed various calcite crystal structures in dry moonmilk (Figures 6A–D) and wet moonmilk (Figures 6E–H). Although both moonmilk samples showed a subhedral calcite crystal with irregular crystal faces (Figures 6A,E), the overall calcite fabrics represented prismatic crystals in dry moonmilk (Figure 6A) and trigonal textures in wet moonmilk (Figure 6E). Additionally, filamentous and spheroidal structures were observed (Figures 6B–D,G,H). Dry moonmilk specimens included several curved filamentous components with a length of few 1000s of micrometers and a width of 1–3 100s of micrometers (Figure 6B). Many spheroidal grains were found in dry moonmilk and some materials stuck around these structures (Figures 6C,D). Recent studies of microbial mats in limestone caves showed that round-shaped microstructures were present in various microbial mats (Riquelme et al., 2015; Cao et al., 2016). These structures were similar to bacteria in size, suggesting that bacterial activity has the potential to affect the formation of these round-shaped structures. In wet moonmilk, we observed many microfibers, filamentous structures, and their aggregates, which were not found in dry moonmilk. These structures were randomly entangled rather than forming in a specific direction (Figures 6G,H).

Although it remains difficult to connect the morphology of calcite structure to microbial activities, we found that the two types of moonmilk have different structures on their surface based on SEM analysis.

## Discussion

Deep areas of a cave are oligotrophic, with constrained light energy and nutrient supplementation, with many factors limiting the survival of microorganisms. Even in such conditions, microbial communities can influence the formation of moonmilk, which can be observed by measuring the metabolic activity and pH changes by nanorespirometry (Sanchez-Moral et al., 2012). In this study, we focused on detecting the presence and diversity of microbial communities using NGS of 16S rRNA genes for bacteria and ITS2 regions for fungi and analyzing the surfaces of two moonmilk samples via SEM. The surface analyses revealed numerous differences in calcite structures between dry and wet moonmilks. Dry moonmilk typically showed spheroidal structures, whereas wet moonmilk contained clusters of filamentous structures, which are thought to be microbial precipitates that may be induced or calcified by different microorganisms.

Metagenomic analysis was used to analyze the microbial communities in the two types of moonmilk compared to those in outside soil. Moonmilk contains several bacteria, such as *Arthrobacter, Rhodobacterales*, and *Xanthomonadales* groups, known to precipitate CaCO_3_ (Cacchio et al., 2003; Anbu et al., 2016). Dry and wet moonmilks showed a significant difference in the microbial community structure. Purple sulfur bacteria *Chromatiales* were the most abundant in dry moonmilk, but were rarely detected in the other samples. A representative sequence of the OTU of *Chromatiales* was found to be identical to that of bacteria in a limestone cave in Spain and Portugal (Riquelme et al., 2015). Previous studies also reported the phylotype of *Chromatiales* to be one of the core microbiomes in limestone caves (Porca et al., 2012; Engel, 2015). Because the same phylotype occupies the major microbial community in geographically distant caves, this phylotype may be specific to limestone caves, and the condition of dry moonmilk appears to be appropriate for the growth of this bacterium. Additionally, *Streptomyces* were found in approximately 1.5% (DM1: 1.04%, DM2: 1.68%, DM3: 1.85%, DM, dry moonmilk) of dry moonmilk samples but not in wet moonmilk samples (Table 3). Secondary metabolites produced by Actinomycetes may directly inhibit or promote the growth of other microorganisms (Patin et al., 2017) and may act as cell-signaling molecules that play important roles in microbial community maintenance (Yim et al., 2007). With the ability to inhibit the growth of other bacteria, the secondary metabolites of *Streptomyces* may have affected the surrounding microorganisms and may explain the different bacterial community composition between dry and wet moonmilk.

Calcite structure of moonmilk exhibit not only nanograins (Figure 6B) due to the classical pathway based on ion-by-ion accumulation (Zhu et al., 2016) but also irregular aggregation-based crystallization (Figures 6A,E–G). Non-classical calcite growth is known to involve organic substances which can stabilize precursor nanoparticles to provide building blocks for aggregation-based crystallization (Zhu et al., 2016). In our SEM results, most of the structure appears to be nanofibers derived from the classical pathway. Still, we can find inorganic crystallization that cannot be produced without the involvement of organic matter in both dry and wet moonmilk. The filamentous structures in moonmilk (Figure 6F) are expected to have been formed by calcified fungal mycelia based on both structure and EDS result (Table 4), because fungi are known to induce CaCO_3_ precipitations composed of needle-fiber calcite and nanofibers (Bindschedler et al., 2016). In ongoing studies, we observed antimicrobial and urease activities of *Streptomyces* species isolated from dry moonmilk. Therefore, we suggest that *Streptomyces* may induce differences in the composition of the microbial community between dry and wet moonmilk and could have a potential role in the formation of spheroidal calcite structures in moonmilk.

**TABLE 4:** EDS analysis revealed the CaCO_3_ composition of filamentous and round-shaped structures.

Spectrum location	Element							
	C		O		Ca		F	
	Weight%	Atomic%	Weight%	Atomic%	Weight%	Atomic%	Weight%	Atomic%
B	12.59	24.98	25.74	38.34	61.67	36.38	–	–
C	5.76	12.56	35.83	58.63	35.45	23.16	1.9	2.62
G	6.47	14.13	35.92	58.94	31.07	20.35	2.28	3.15
H	11.78	18.6	55.66	65.99	32.56	15.41	–	–

As for wet moonmilk, two sample-specific phylotypes, *Xanthomonadales* and *Arthrobacter*, were observed among OTUs with high abundance. These bacteria have been reported to play a role in calcite precipitation and the presence of water may be responsible for the different types of bacteria acting on calcite precipitation (Baskar et al., 2016; Montano-Salazar et al., 2018). We also found that *Pseudomonas* and *Streptococcus* were dominant in one of the wet moonmilk samples. These bacteria are known to play a major role in biofilm formation and the carbonate bio-mineralization therein (Li et al., 2015). Calcite precipitation following biofilm formation likely contributed to the specific structure of wet moonmilk observed in SEM images in this study.

Among the OTUs constituting the microbial communities of moonmilk, common OTUs for bacteria such as *Acidimicrobiales* and *Rhizobiales* are shared in the outside soil near the entrance of the cave. Some microbial communities in the cave were likely influenced by sources outside the cave. This phenomenon was also found in other caves, and similar patterns of community composition have been reported for various sites in the cave (Wu et al., 2015). Particularly, *Rhizobiales*, found in the outside soil samples, is an order associated with nitrogen-fixation (Carvalho et al., 2010), suggesting that the moonmilk microbial communities were transferred from the soil. This is supported by the fact that the fungus *Mortierella* was exceptionally dominant in both outside soil and moonmilk. These results indicate that microbial ecosystems of the cave are supplied with energy sources such as nitrogen from external soil environments. As *Mortierella* is usually found in soil, it may have flowed into the cave in the past. In addition, the oligotrophic condition may provide a tough environment in which other fungi in outside soil samples cannot survive in the cave. Further study is needed on the microbial ecology in speleothem as well as moonmilk to confirm whether the cave is affected by its external environment, although it is considered a restricted ecosystem.

## Conclusion

We revealed several traces predicted as microbial structures in two types of moonmilk by SEM. Analysis of the microbial community of moonmilk using NGS showed that different calcite-forming bacteria were present in the two types of moonmilk. Taken together, these findings suggest that moonmilk from the Baeg-nyong cave can have a microbial community structure that differs from that of the outside soil. Furthermore, specific microorganisms may cooperate to form various calcite structures depending on the state of moonmilk.

## Data Availability Statement

The datasets generated and/or analyzed during the current study are available in the Sequence Read Archive (SRA), hosted by the National Center for Biotechnology Information (NCBI), available with accession number PRJNA588777.

## Author Contributions

J-SL designed the research. SP, Y-JC, DJ, and KJ performed the research. E-JL provided expertise. J-SL, SP, and Y-JC wrote the manuscript.

## Conflict of Interest

The authors declare that the research was conducted in the absence of any commercial or financial relationships that could be construed as a potential conflict of interest.

## Supplementary Material

TABLE S1 Total number of bacterial OTUs from the whole sample.

TABLE S2 Total number of fungal OTUs from the whole sample.

## References

[B1] Akob D. M. Kusel K. (2011). Where microorganisms meet rocks in the Earth’s Critical Zone. *Biogeosciences* 8 3531–3543. 10.5194/bg-8-3531-2011

[B2] Anbu P. Kang C. H. Shin Y. J. So J. S. (2016). Formations of calcium carbonate minerals by bacteria and its multiple applications. *Springerplus* 5:250. 10.1186/s40064-016-1869-2 27026942 PMC4771655

[B3] Barnard R. L. Osborne C. A. Firestone M. K. (2013). Responses of soil bacterial and fungal communities to extreme desiccation and rewetting. *ISME J.* 7 2229–2241. 10.1038/ismej.2013.104 23823489 PMC3806258

[B4] Barton H. A. (2006). Introduction to cave microbiology: a review for the non-specialist. *J. Cave Karst Stud.* 68 43–54.

[B5] Barton H. A. Northup D. E. (2007). Geomicrobiology in cave environments: past, current and future perspectives. *J. Cave Karst Stud.* 69 163–178.

[B6] Baskar S. Routh J. Baskar R. Kumar A. Miettinen H. Itavaara M. (2016). Evidences for microbial precipitation of calcite in Speleothems from Krem Syndai in Jaintia Hills, Meghalaya, India. *Geomicrobiol. J.* 33 906–933. 10.1080/01490451.2015.1127447

[B7] Bindschedler S. Cailleau G. Verrecchia E. (2016). Role of fungi in the biomineralization of calcite. *Minreals* 6:41 10.3390/min6020041

[B8] Bolger A. M. Lohse M. Usadel B. (2014). Trimmomatic: a flexible trimmer for Illumina sequence data. *Bioinformatics* 30 2114–2120. 10.1093/bioinformatics/btu170 24695404 PMC4103590

[B9] Borsato A. Frisia S. Jones B. Borg K. V. D. (2000). Calcite Moonmilk: crystal morphology and environment of formation in caves in the Italian alps. *J. Sediment. Res.* 70 1171–1182. 10.1306/032300701171

[B10] Cacchio P. Ercole C. Cappuccio G. Lepidi A. (2003). Calcium carbonate precipitation by bacterial strains isolated from a limestone cave and from a loamy soil. *Geomicrobiol. J.* 20 85–98. 10.1002/jobm.201300560 25590872

[B11] Cacchio P. Ercole C. Cappuccio G. Lepidi A. (2012). Biogenicity and characterization of moonmilk in the grotta nera (majella national park, abruzzi, central Italy). *J. Cave Karst Stud.* 76 88–103. 10.4311/2012mb0275

[B12] Cañaveras J. C. Cuezva S. Sanchez-Moral S. Lario J. Laiz L. Gonzalez J. M. (2006). On the origin of fiber calcite crystals in moonmilk deposits. *Naturwissenschaften* 93 27–32. 10.1007/s00114-005-0052-3 16240102

[B13] Cañaveras J. C. Hoyos M. Sanchez-Moral S. Sanz-Rubio E. Bedoya J. Soler V. (1999). Microbial communities associated with hydromagnesite and needle-fiber aragonite deposits in a karstic cave (Altamira, northern Spain). *Geomicrobiol. J.* 16 9–25. 10.1080/014904599270712

[B14] Cao C. Jiang J. Sun H. Huang Y. Tao F. Lian B. (2016). Carbonate mineral formation under the influence of limestone-colonizing Actinobacteria: morphology and polymorphism. *Front. Microbiol.* 7:366. 10.3389/fmicb.2016.00366 27148166 PMC4834437

[B15] Carvalho F. M. Souza R. C. Barcellos F. G. Hungria M. Vasconcelos A. T. R. (2010). Genomic and evolutionary comparisons of diazotrophic and pathogenic bacteria of the order Rhizobiales. *BMC Microbiol.* 10:37. 10.1186/1471-2180-10-37 20144182 PMC2907836

[B16] Cirigliano A. Tomassetti M. C. Pietro M. D. Mura F. Maneschi M. L. Gentili M. D. (2018). Calcite moonmilk of microbial origin in the Etruscan Tomba degli Scudi in Tarquinia, Italy. *Sci. Rep.* 8:15839. 10.1038/s41598-018-34134-y 30367083 PMC6203712

[B17] Curry M. D. Boston P. J. Spilde M. N. Baichtal J. F. Campbell A. R. (2009). Cottonballs, a unique subaqeous moonmilk, and abundant subaerial moonmilk in Cataract Cave, Tongass National Forest, Alask. *Int. J. Speleol.* 38 111–128. 10.5038/1827-806x.38.2.3

[B18] Edgar R. C. Haas B. J. Clemente J. C. Quince C. Knight R. (2011). UCHIME improves sensitivity and speed of chimera detection. *Bioinformatics* 27 2194–2200. 10.1093/bioinformatics/btr381 21700674 PMC3150044

[B19] Engel A. S. (2015). “Methods for characterizing microbial communities in caves and Karst: a review,” in *Microbial Life of Cave Systems Life in Extreme Environments*, Vol. VOL. 3, ed. Engel A. S. (Berlin: Walter de Gruyter GmbH & Co. KG), 23–46.

[B20] Garcia-Sanchez A. Machado-Moreira B. Freire M. Santos R. Monteiro S. Dias D. (2019). Characterization of microbial communities associated with ceramic raw materials as potential contributors for the improvement of ceramic rheological properties. *Minerals* 9:316 10.3390/min9050316

[B21] Herlemann D. P. Labrenz M. Jürgens K. Bertilsson S. Waniek J. J. Andersson A. F. (2011). Transitions in bacterial communities along the 2000 km salinity gradient of the Baltic Sea. *ISME J.* 5 1571–1579. 10.1038/ismej.2011.41 21472016 PMC3176514

[B22] Hill C. A. Forti P. (2007). Cave mineralogy and the NSS: Past, present, future. *J. Cave Karst Stud.* 69 35–45.

[B23] Ihrmark K. Bödeker I. T. Cruz-Martinez K. Friberg H. Kubartova A. Schenck J. (2012). New primers to amplify the fungal ITS2 region–evaluation by 454-sequencing of artificial and natural communities. *FEMS Microbiol. Ecol.* 82 666–677. 10.1111/j.1574-6941.2012.01437.x 22738186

[B24] Jung D. Y. Park S. Lee J. S. Jo K. N. (2017). Microbial structures and their distributions in cave tufa formations from a twilight zone of the Baeg-nyong Cave. *J. Geol. Soc. Korea* 53 743–758. 10.14770/jgsk.2017.53.5.743

[B25] Kim M. Kim W. Tripathi B. M. Adams J. M. (2014). Distinct bacterial communities dominate tropical and temperate zone leaf litter. *Microb. Ecol.* 67 837–848. 10.1007/s00248-014-0380-y 24549745

[B26] Kim O. S. Cho Y. J. Lee K. Yoon S. H. Kim M. Na H. (2012). Introducing EzTaxon-e: a prokaryotic 16S rRNA gene sequence database with phylotypes that represent uncultured species. *Int. J. Syst. Evol. Microbiol.* 62 716–721. 10.1099/ijs.0.038075-0 22140171

[B27] Li X. Chopp D. L. Russin W. A. Brannon P. T. Parsek M. R. Packman A. I. (2015). Spatial patterns of carbonate biomineralization in biofilms. *Appl. Environ. Microbiol.* 81 7403–7410. 10.1128/AEM.01585-15 26276112 PMC4592860

[B28] Macalady J. Dattagupta S. Schaperdoth I. Jones D. S. Druschel G. K. Eastman D. (2008). Niche differentiation among sulfur-oxidizing bacterial populations in cave waters. *ISME J.* 2 590–601. 10.1038/ismej.2008.25 18356823

[B29] McMurdie P. J. Holmes S. (2013). phyloseq: an R package for reproducible interactive analysis and graphics of microbiome census data. *PLoS One* 8:e61217. 10.1371/journal.pone.0061217 23630581 PMC3632530

[B30] Montano-Salazar S. M. Lizarazo-Marriaga J. Brandao P. F. B. (2018). Isolation and potential biocementation of calcite precipitation inducing bacteria from Colombian buildings. *Curr. Microbiol.* 75 256–265. 10.1007/s00284-017-1373-0 29043388

[B31] Northup D. E. Lavoie K. H. (2001). Geomicrobiology of caves: a review. *Geomicrobiol. J.* 18 199–222. 10.1080/01490450152467750

[B32] Ortiz M. Neilson J. W. Nelson W. M. Legatzki A. Byrne A. Yu Y. (2013). Profiling bacterial diversity and taxonomic composition on speleothem surfaces in Kartchner Caverns, AZ. *Microb. Ecol.* 65 371–383. 10.1007/s00248-012-0143-6 23224253

[B33] Patin N. V. Schorn M. Aguinaldo K. Lincecum T. Moore B. S. Jensen P. R. (2017). Effects of actinomycete secondary metabolites on sediment microbial communities. *Appl. Environ. Microbiol.* 83:e02676-16. 10.1128/AEM.02676-16 27986719 PMC5288833

[B34] Porca E. Jurado V. Zgur-Bertok D. Saiz-Jimenez C. Pasic L. (2012). Comparative analysis of yellow microbial communities growing on the walls of geographically distinct caves indicates a common core of microorganisms involved in their formation. *FEMS Microbiol. Ecol.* 81 255–266. 10.1111/j.1574-6941.2012.01383.x 22486654

[B35] Portillo M. C. Gonzalez J. M. (2009). Sulfate-reducing bacteria are common members of bacterial communities in Altamira Cave (Spain). *Sci. Total Environ.* 407 1114–1122. 10.1016/j.scitotenv.2008.10.045 19027143

[B36] Portillo M. C. Gonzalez J. M. (2011). Moonmilk deposits originate from specific bacterial communities in Altamira Cave (Spain). *Microb. Ecol.* 61 182–189. 10.1007/s00248-010-9731-5 20717660

[B37] Riquelme C. Rigal F. Hathaway J. J. Northup D. E. Spilde M. N. Borges P. A. (2015). Cave microbial community composition in oceanic islands: disentangling the effect of different colored mats in diversity patterns of Azorean lava caves. *FEMS Microbiol. Ecol.* 91:fiv141. 10.1093/femsec/fiv141 26564959

[B38] Rooney D. C. Hutchens E. Clipson N. Baldini J. McDermott F. (2010). Microbial community diversity of moonmilk deposits at Ballynamintra Cave, Co. Waterford, Ireland. *Microb. Ecol.* 60 753–761. 10.1007/s00248-010-9693-7 20567814

[B39] Rutherford J. M. Huang L. H. (1994). A study of fungi of remote sediments in West Virginia caves and a comparison with reported species in the literature. *NSS Bull.* 56 38–45.

[B40] Sanchez-Moral S. Portillo M. C. Janices I. Cuezva S. Fernández-Cortés A. Cañaveras J. C. (2012). The role of microorganisms in the formation of calcitic moonmilk deposits and speleothems in Altamira Cave. *Geomorphology* 139 285–292. 10.1016/j.geomorph.2011.10.030

[B41] Schloss P. D. Westcott S. L. Ryabin T. Hall J. R. Hartmann M. Hollister E. B. (2009). Introducing mothur: open-source, platform-independent, community-supported software for describing and comparing microbial communities. *Appl. Environ. Microbiol.* 75 7537–7541. 10.1128/AEM.01541-09 19801464 PMC2786419

[B42] Segata N. Izard J. Waldron L. Gevers D. Miropolsky L. Garrett W. S. (2011). Metagenomic biomarker discovery and explanation. *Genome Biol.* 12:R60. 10.1186/gb-2011-12-6-r60 21702898 PMC3218848

[B43] Westcott S. L. Schloss P. D. (2017). OptiClust, an improved method for assigning amplicon-based sequence data to operational taxonomic units. *mSphere* 2:e00073-17. 10.1128/mSphereDirect.00073-17 28289728 PMC5343174

[B44] Woo K. S. Kim R. Lee K. C. Choi Y. G. Choi D. W. (2006). *Scientific Investigation of the Baegnyong Cave, Pyeongchanggun, Gangwondo.* Pyeongchanggu: Cultural Heritage Administration (CHA), 222.

[B45] Wu Y. Tan L. Liu W. Wang B. Wang J. Cai Y. (2015). Profiling bacterial diversity in a limestone cave of the western Loess Plateau of China. *Front. Microbiol.* 6:244. 10.3389/fmicb.2015.00244 25870592 PMC4378288

[B46] Yang S. Zhang Y. Cong J. Wang M. Zhao M. Lu H. (2017). Variations of soil microbial community structures beneath broadleaved forest trees in temperate and subtropical climate zones. *Front. Microbiol.* 8:200. 10.3389/fmicb.2017.00200 28239373 PMC5300970

[B47] Yim G. Wang H. H. Davies J. (2007). Antibiotics as signalling molecules. *Philos. Trans. R. Soc. B* 362 1195–1200. 10.1098/rstb.2007.2044 17360275 PMC2435582

[B48] Zhang J. Kobert K. Flouri T. Stamatakis A. (2014). PEAR: a fast and accurate Illumina Paired-End reAd mergeR. *Bioinformatics* 30 614–620. 10.1093/bioinformatics/btt593 24142950 PMC3933873

[B49] Zhu G. Yao S. Zhai H. Liu Z. Li Y. Pan H. (2016). Evolution from classical to non-classical aggregation-based crystal growth of calcite by organic additive control. *Langmuir* 32 8999–9004. 10.1021/acs.langmuir.6b01594 27519793

[B50] Zhu T. Dittrich M. (2016). Carbonate precipitation through microbial activities in natural environment, and their potential in biotechnology: a review. *Front. Bioeng. Biotechnol.* 4:4. 10.3389/fbioe.2016.00004 26835451 PMC4718973

